# Nutrigenetics and Omega-3 and Gamma-Linolenic Acid Intake and Status in Patients with Cancer: A PRISMA Scoping Review of Research Trends and Challenges

**DOI:** 10.3390/ijms26104867

**Published:** 2025-05-19

**Authors:** Vladica Zikic, Marija Paunovic, Marijana Milovic-Kovacevic, Vesna Vucic, Danijela Ristic-Medic

**Affiliations:** 1 Cognitive Neuroscience Department, Research and Development Institute “Life Activities Advancement Institute”, 11000 Belgrade, Serbia; vladica.zikic@gmail.com; 2 Department of Speech, Language and Hearing Sciences, Institute for Experimental Phonetics and Speech Pathology, 11000 Belgrade, Serbia; 3 Group for Nutritional Biochemistry and Dietology, Centre of Research Excellence in Nutrition and Metabolism, Institute for Medical Research, National Institute of Republic of Serbia, University of Belgrade, 11000 Belgrade, Serbia; marija.paunovic@imi.bg.ac.rs (M.P.); danijelar@imi.bg.ac.rs (D.R.-M.); 4 Department of Medical Oncology, Institute of Oncology and Radiology Serbia, 11000 Belgrade, Serbia; marijana.milovic.kovacevic@gmail.com; 5 Faculty of Medicine, University of Belgrade, 11000 Belgrade, Serbia

**Keywords:** fatty acid desaturase, *FADS1*, *FADS2*, omega-3 fatty acids, gamma-linolenic acid, single-nucleotide polymorphisms, cancer, cancer risk

## Abstract

Epidemiological studies report inconsistent findings regarding the association between dietary polyunsaturated fatty acid (PUFA) intake and cancer risk. Genetic variations—particularly single-nucleotide polymorphisms (SNPs) in the *FADS1* and *FADS2* genes—affect PUFA metabolism, linking circulating PUFA levels to the risk of several cancers, including breast, colorectal, prostate, and pancreatic cancers. This review aimed to investigate the relationship between *FADS1* and *FADS2* gene variants and dietary intake, supplementation, or intervention with omega-3 fatty acids, gamma-linolenic acid (GLA), or their combination in cancer patients. A secondary objective was to examine genetically determined fatty acid profiles—shaped by *FADS1* and *FADS2* polymorphisms—in cancer patients without intervention and their potential association with PUFA-related cancer risk. A systematic search of the Scopus, PubMed, and Web of Science databases (up to 2024) identified 11 eligible studies out of 298 initial records. Analysis of the available literature suggests that specific *FADS* genotypes influence long-chain PUFA (LC-PUFA) concentrations in blood and tissues and that altered LC-PUFA levels may contribute to cancer development. The most consistent association identified is between the rs174537 variant and altered PUFA metabolism in prostate and breast cancer. However, conclusive evidence is lacking on the impact of dietary patterns on FADS desaturase activity or expression. Only one study has examined omega-3 supplementation in relation to *FADS* gene variants in prostate cancer patients, while the effects of GLA supplementation remain unexplored. Given the relative novelty of this research area and the limited number of studies, future investigations should integrate dietary PUFA intake, genetic variation in PUFA-metabolizing enzymes, and potential gene–nutrient interactions involving *FADS* gene polymorphisms and PUFAs to clarify their role in cancer risk.

## 1. Introduction

**Figure 1:**
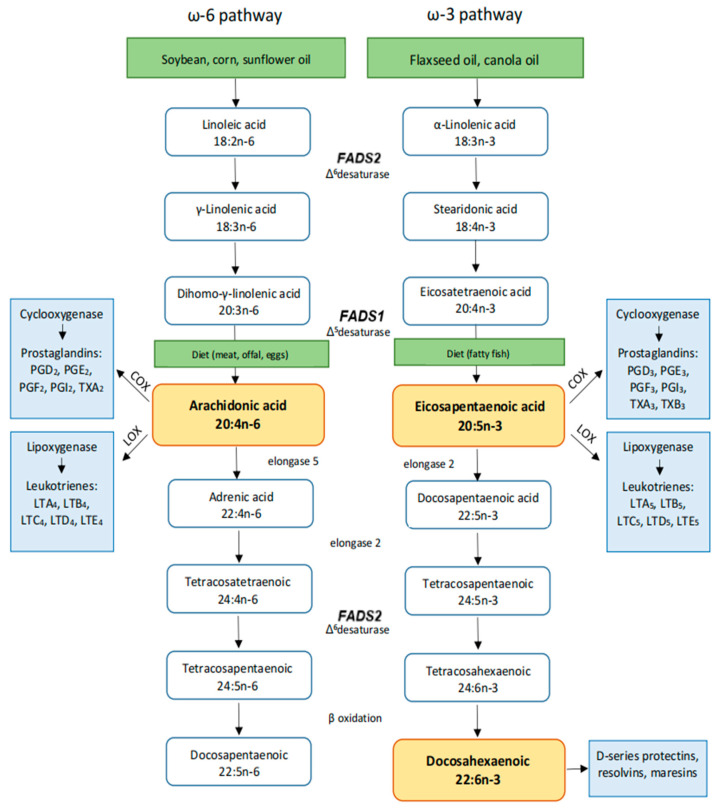
Pathways of long-chain omega-6 and omega-3 fatty acid synthesis from dietary intake and their further metabolism to the production of inflammation mediators: involvement of *FADS1* and *FADS2* genes.

Cancer represents the second leading cause of death globally [1]. Projections suggest that by the end of the century it will become the leading cause of premature death and the primary factor limiting lifespan [2]. A diet rich in vegetables, whole grains, and healthy fats, enriched with antioxidants, may reduce cancer risk [3,4]. Nutrigenetics explores how genetic variations influence the body’s response to diet, shaping nutrient metabolism, dietary effects, and the risk of diet-related illnesses [5,6]. By considering an individual’s genetic makeup, nutrigenetics aims to develop personalized nutrition, enabling more effective and targeted dietary recommendations [7]. Dietary fats play essential roles in different physiological processes, impacting plasma fatty acid profiles. Their metabolism involves the delta-5 and delta-6 desaturase enzymes encoded by the fatty acid desaturase 1 (*FADS1*) and desaturase 2 (*FADS2*) genes, located on human chromosome 11 (11q12-13.1). These enzymes are involved in the endogenous conversion process of 18-carbon polyunsaturated fatty acids (PUFAs) into long-chain PUFAs (LC-PUFAs) such as arachidonic acid (AA), docosahexaenoic acid (DHA), and eicosapentaenoic acid (EPA) [8,9,10,11] (Figure 1).

Variations in single-nucleotide polymorphisms (SNPs) within the *FADS* genes significantly influence an individual’s ability to synthesize EPA and DHA [9], thereby affecting LC-PUFA levels. This modulation of lipid metabolism may play a crucial role in the onset of different diet-related disorders. LC-PUFAs also serve as precursors for eicosanoids, bioactive molecules that regulate inflammation through either pro-inflammatory or anti-inflammatory pathways [12]. Chronic inflammation is a well-established risk factor for numerous diseases, including neurodegenerative diseases, type 2 diabetes, cardiovascular disease, and various cancers [13]. Despite being an omega-6 PUFA, gamma-linolenic acid (GLA) exerts anti-inflammatory effects via its conversion to dihomo-gamma-linolenic acid (DGLA) and subsequently to prostaglandin PGE1. Among LC-PUFAs, AA is primarily a precursor for pro-inflammatory eicosanoids, and its plasma phospholipid concentration may contribute to the development of certain cancers [14]. In contrast, omega-3 LC-PUFAs, such as EPA and DHA, exhibit anti-inflammatory properties and have been associated with a protective effect against the development of breast [15], colorectal [16], and pancreatic cancers [17].

Cancer tissue also alters lipid metabolism compared to healthy tissue, notably by increasing AA levels [18], a process that may be influenced by genetic variation within the *FADS* locus [19]. It was shown that the efficiency of LC-PUFA synthesis was highly dependent on the rs174537 genotype. Individuals with the GG genotype had higher concentrations of LC-PUFAs (both omega-6 and omega-3) compared to those with the TT genotype [20]. On the other hand, studies have confirmed that the increased activity of PUFA desaturases, associated with the rs174546 genetic variant, correlates with a higher risk of developing lung cancer, different subtypes of colorectal cancer, esophageal squamous cell carcinoma, respiratory and intrathoracic cancers, basal cell carcinoma, and non-melanoma and overall skin cancer [21]. Similar data have been collected for rs174548 variants, where their presence has been linked to an increased risk of lung cancer [22]. Beyond the blood, expression of *FADS1* and *FADS2* genes has been documented in at least 44 different tissues, with the highest level of expression in the adrenal gland and brain tissue [19].

Even though *FADS* polymorphisms can influence fatty acid profiles [23], data on the relationship between nutrition enriched with LC-PUFAs and changes in their expression remain inconclusive. While a study on personalized omega-3 dosages showed an increase in their blood levels, there is insufficient evidence to determine whether these variations result from *FADS1/2* gene variants or personalized supplementation [24]. Additionally, the prevalence of the TT polymorphism in the rs174583 variant of the *FADS2* gene has been associated with a higher risk of obesity and increased body mass index in carriers. However, research has not demonstrated a clear correlation between these outcomes and different dietary patterns [25]. FADS1 expression has been linked to cancer progression. It is overexpressed in bladder tumors, correlating with higher tumor grade and enhanced proliferation [26], whereas its downregulation in non-small-cell lung cancer and bladder cancer is associated with tumor characteristics (location, size, and histological grade) and poor prognosis [27]. FADS enzymes also influence inflammation, DNA repair, and apoptosis, and they have been associated with distinct molecular subtypes of tumors in breast cancer. These findings suggest a strong correlation between FADS2 expression and clinicopathological characteristics, highlighting its potential as a diagnostic biomarker for breast cancer [28]. Moreover, according to available data, FADS1 is causally involved in cancer cell proliferation and, together with FADS2, has emerged as a promising pharmacological target for anti-cancer therapy [26,29,30,31]. Elevated FADS1 expression is associated with poor prognosis, tumor progression, and an altered tumor microenvironment. Patients with high FADS1 expression may benefit from FADS1-targeted therapies, as inhibition has been shown to suppress cancer cell growth. Similarly, FADS2 is highly expressed in breast cancer, and its knockout reduces cell invasion, migration, and colony formation. These findings suggest that targeting FADS1/2, particularly when combined with genome-tailored nutritional strategies, could offer a novel and effective approach for precision oncology in selected patient groups.

Since the data on PUFA metabolism and *FADS* polymorphisms remain inconclusive, this review aimed to examine the influence of *FADS1* and *FADS2* gene variants on metabolism and biological effects of omega-3 fatty acids and gamma-linolenic acid, both individually and in combination, in cancer patients. This included evaluating the impact of dietary intake, supplementation, and intervention, as well as assessing the genetically determined status of PUFAs in relation to cancer risk, disease progression, and treatment outcomes, across different cancer types. To the best of our knowledge, no previous review has focused on this specific combined assessment.

## 2. Results

**Figure 2:**
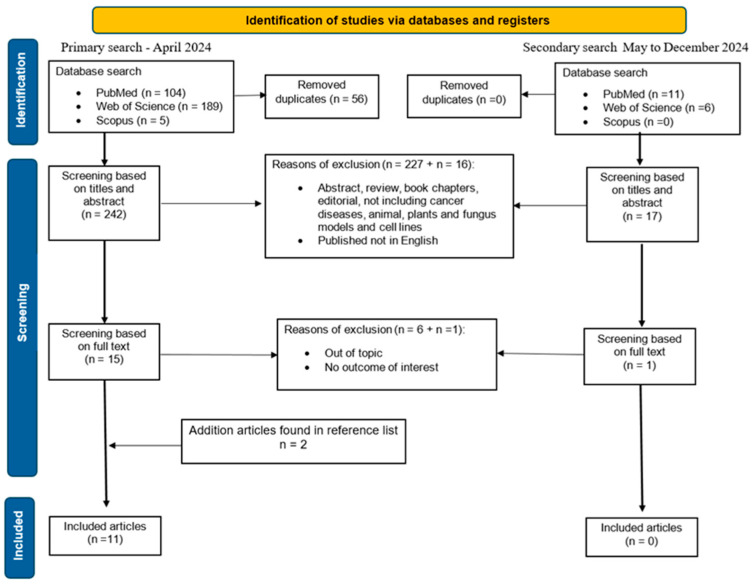
PRISMA flowchart of the search process. *n* = mean number of articles.

A total of *n* = 298 articles were retrieved in the initial search from all three databases, covering the period up to the end of April 2024. After removing duplicates (*n* = 56), a total of *n* = 242 articles were screened based on the title and abstract. Of those, *n* = 227 were excluded (abstracts, reviews, editorials, book chapters, and publications not in English or on irrelevant topics). The remaining 15 articles underwent a full review, of which 4 were excluded (irrelevant topic or endpoints). Two more were added from the reference lists. Finally, *n* = 11 articles met the eligibility criteria and were evaluated in this review. To update the search, we conducted secondary research of databases, covering the period from the beginning of May until the end of 2024. Out of 17 new articles, none fit the criteria; hence, the final number of studies remained 11. Figure 2 presents the PRISMA flowchart of the study selection process. Notably, most of the included studies were published between 2017 and 2024, with only two articles from 2012 and 2013. The relative novelty of this research area explains the limited number of available studies. The selected articles were divided into three groups: 1. dietary intervention with omega-3 PUFAs, along with genetic variants of *FADS1/FADS2* genes, in cancer patients (only one study, Table 1); 2. PUFA status in plasma/tissues and its association with *FADS1/FADS2* polymorphism in cancer patients (three studies, Table 2); and 3. PUFA status in relation to *FADS1/FADS2* genotypes as risk factors for cancer development (7 studies, Table 3).

Most studies were conducted in the USA (five studies), followed by China (two studies), while Poland, India, and Korea each contributed one study. Additionally, one study covered both the USA and Ghana (Table 1, Table 2 and Table 3). Among the selected studies, only one [32] investigated a dietary intervention in prostate cancer patients using a flaxseed-enriched diet. Three studies focused on PUFA status and genetic variation in *FADS1/FADS2* genes, two on prostate cancer [33,34] and one on glioblastoma multiforme [35]. The remaining seven studies examined the relationship between PUFA status, SPNs in *FADS* genes, and cancer risk factors [36,37,38,39,40,41,42].

Regarding PUFA measurement methods, three studies analyzed PUFA status from whole blood [33,38,41], while four studies measured PUFAs in malignant tissue [32,34,36,39] and in both tumor tissues and blood components (red blood cells/serum) [37,40]. Among the studies that did not directly assess PUFA levels, one estimated PUFA intake using a Food Frequency Questionnaire (FFQ) [42], while the other focused on mRNA expression levels of desaturase enzymes [35].

**Table 1:** Dietary supplementation of omega-3 fatty acids, along with genetic variants in the *FADS1/FADS2* gene clusters, in cancer patients.

Reference	Country	Cancer Area	Study Design	Sample Size, (*n*)	Age (Years)	Genetic Variation in the *FADS1/FADS2* Gene Cluster	Dietary Assessment	Intervention/ Supplementation Duration	PUFA Status	Study-Related Conclusions
Azrad et al., 2012 [32]	USA	Prostate cancer	RCT	*n* = 161 I-control (*n* = 41), II-flaxseed (FS) (*n* = 40)	C-59 (36-71) FS-60 (44-73)	rs99780, rs174537, rs174545, rs174572, rs498793, rs3834458, rs968567	NCI DHQ ALA intake: 1.23 vs. 7.57 g/day C vs. FS	Flaxseed (30 g/day) 30 days follow-up	Prostatic tissue	− Dietary intake: ↑ ALA; ↑ ω-3:ω-6 ratio in FS − Prostate levels: ALA similar, ↑ EPA in FS − rs498793 and ALA: significant interaction and influence on aggressive prostatecancer biomarkers independent of the amount of ALA consumed

Abbreviations: RCT, Randomized Control Trial; NCI DHQ, National Cancer Institute Diet History Questionnaire; ALA, Alpha-Linolenic Acid; EPA, Eicosapentaenoic Acid, *n* = mean number.

**Table 2:** PUFA status in plasma/tissues and its association with *FADS1/FADS2* polymorphism in cancer patients.

Reference	Country	Cancer Area	Study Design	Sample Size, *n*, Sex (%)	Age (Mean ± SD or Range)	Genetic Variation in the *FADS1/FADS2* Gene Cluster	Dietary Assessment	PUFA Status: Blood/ Tissues	Associations Between *FADS1* and *FADS2* Genotypes	Study-Related Conclusions
Minas et al., 2023 [33]	Ghana, USA	Prostate Cancer	Case–control	*n* = 976; male -*n* = 489 African American *n* = 487 European American; 1033 controls, 485 African American and 548 European American	50–74 years	rs174556	Nutritional questionnaire	Whole blood	SNP significant associations with ω-6 FA (AA, DGLA, ADA, GLA) in European American men; exception: rs174556 SNP in *FADS1* gene; SNPs did not influence ω-6 levels in African American or Ghanaian men	DHA, DPA, and EPA: inversely associated with prostate cancer among Ghanaian men − ω-6 FAs associated with prostate cancer among European American men − *trans* FAs positively associated with prostate cancer in all study population Palmitoleic acid: positive dose-dependent relationship with higher NCCN risk scores
Korbecki et al., 2020 [35]	Poland	Glioblastoma multiforme	Case–control	*n* = 28 (16 males, 12 females)	60 ± 12 years	Quantitative determination of total FADS, FADS2 expression	No	mRNA expression levels of desaturase enzymes SCD and FADS2	FADS1 and FADS2 expression ↓ in growing tumor area and necrotic core vs. peritumoral area FADS2 expression in the peritumoral area: 2 times higher than in the necrotic core Expression of desaturases in GBM tumors does not differ between the sexes	Biosynthesis of MUFAs and PUFAs in GBM tumors is less intense than in the peritumoral area Nutritional deficiency increases the biosynthesis of MUFAs and PUFAs in GBM cells
Cui et al., 2016 [34]	USA	Prostate cancer	C-S	*n* = 60 55 European American, 4 African American, and 1 Asian	No data	rs174537	No	Prostate tissue	G allele at rs174537: ↑ levels of AA and ADA ↑ ω-3 LC-PUFAs (DHA, DPA) and more efficient *n*-6 PUFA biosynthesis (higher AA/LA and AA/DGLA ratios) FADS1 activity: higher in G allele carriers	AA: 15.8% of total FAs; ω-6 PUFA pathway in specimens from homozygous G individuals exhibited increasingly higher values vs. heterozygous and homozygous T individuals Efficient ω -6 PUFA biosynthesis: promotes tumor growth via lipid signaling Higher AA levels in homozygous GG may influence PCA

FADS, Fatty Acid Desaturase; SNP, Single-Nucleotide Polymorphism; AA, Arachidonic Acid; DGLA, Dihomo-γ-Linolenic Acid; ADA, Adrenic Acid; GLA, Gamma-Linolenic Acid; DHA, Docosahexaenoic Acid; DPA, Docosapentaenoic Acid; EPA, Eicosapentaenoic Acid; C-S, Cross-Sectional Study; NCCN, National Comprehensive Cancer Network; LA, Linoleic Acid; SCD, Stearoyl-CoA Desaturase; GBM, Glioblastoma Multiforme; MUFAs, Monounsaturated Fatty Acids; PUFAs, Polyunsaturated Fatty Acids.

**Table 3:** PUFA status and FADS1/FADS2 genotypes as potential risk factors for cancer development.

Reference	Country	Cancer Type	Study Design	Sample Size, *n*, Sex (%)	Age (Mean + SD/Range)	Genetic Variation in the *FADS1*/*FADS2* Gene Cluster	Dietary Assessment or Intervention	PUFA Status: Blood/ Tissues	Gene–PUFA Interaction and Cancer Risk
White et al., 2019 [40]	Tennessee, USA	Colorectal cancer	RCT	*n* = 141	40 to 80 years	*FADS1* rs174535	No 3 fish oil capsules (1395 mg EPA +1125 mg DHA) 3 olive oil capsules oil (total 3g)	Red blood cells	RBC membrane: − AA lower in homozygous individuals (T allele of the FADS gene) − LA was greater in individuals who had the T allele − No interaction between fish oil supplementation and urinary PGE-M based on FADS genotype − No effect modification between ω-3 LC-PUFA supplementation and urinary PGE-M based on ↑NSAID use − No interaction between fish oil supplementation and rectal eicosanoids based on FADS genotype
Porenta et al., 2013 [37]	California, USA	Colon cancer	RCT	*n* = 108	51.1–54.9	rs3834458, rs174556, rs174561, rs174537	Mediterranean diet group/ healthy eating group baseline and after 6 months 2-day food records and two 24 h recalls	Serum, colon tissue	− AA at 6 months—significantly different between diet arms in persons with no minor alleles in *FADS1/2* gene cluster − Colon AA at 6 months in no minor allele carriers ↑ HE ↑MD MD may reduce colon cancer risk in individuals with no minor alleles
Wang et al., 2017 [39]	China	Lung cancer	OCS/GWAS (observational, cross-sectional genome-wide association study)	*n* = 253		*FADS1* rs174548	No	Liver Tissue	rs174548 Stronger effect on lung cancer risk in females Only mQTL variant of PUFAs reported by previous GWASs and explained a large proportion of heritability Plasma PUFAs causally associated with lung cancer based on the idea of Mendelian randomization
Murff et al., 2021 [36]	Tennessee, USA	Colorectal cancer	RCT	*n* = 141	40-80 years	rs174535	No dietary data Intervention: capsules fish oil (1395 mg EPA + 1125 mg DHA)	RBCs Rectal epithelial cells	*FADS* genotype: No influence on RBC membrane ω-3 LCPUFA percentages in response to supplementation − Impact on RBC membrane AA content decreased − Regardless of the *FADS* genotype, no evidence of a proliferative or pro-apoptotic effect on ω-3 LC-PUFA supplementation on rectal mucosae
Chen et al., 2017 [41]	China	Oral cancer	Case–control	*n* = 305 oral cancer patients; *n* = 579 healthy controls	20 to 80 years	rs174549	Fish intake	Whole blood	− Significant gene–diet multiplicative interaction between *FADS1* rs174549 polymorphism and fish intake for oral cancer *FADS1*—a variant allele associated with a significantly decreased risk of oral cancer AA genotype associated with a decreased risk of oral cancer compared to the GG genotype
Preethika et al., 2022 [38]	India	Breast cancer	OCS	102	25-60 age range	rs 174537	No	Whole blood	− DNA variation: does not lead to cancer; it modifies molecular traits that go on to affect breast cancer risk − High levels of AA and low levels of ω-3 LC-PUFAs − Individuals who exhibit lower FADS1 activity (T allele) benefit from ω-3 LC-PUFAs by reduction in risk of breast cancer
Lee et al., 2018 [42]	Korea	Gastric cancer	Case–control	402 cases 1.062 controls	Cases: 55.27 ± 10.91 Controls: 52.03 ± 8.60	*FADS1* rs174546 *FADS2* rs174583	Semi-quantitative FFQ composed of 106 food items	No	− Inverse association between dietary DHA and the risk of gastric cancer − *FADS1* rs174546 and *FADS2* rs174583: did not change association between ω-3 or ω-6 PUFAs and gastric cancer risk

RCT, Randomized Controlled Trial; FADS, Fatty Acid Desaturase; EPA, Eicosapentaenoic Acid; DHA, Docosahexaenoic Acid; AA, Arachidonic Acid; LA, Linoleic Acid; PGE-M, Prostaglandin E Metabolite; NSAID, Nonsteroidal Anti-Inflammatory Drug; mOTL, Modified Oligonucleotide Therapy; GWAS, Genome-Wide Association Study; PUFAs, Polyunsaturated Fatty Acids; RBCs, Red Blood Cells; LCPUFAs, Long-Chain Polyunsaturated Fatty Acids; FFQ, Food Frequency Questionnaire.

## 3. Discussion

Although diet can be a contributing factor in the development of various chronic diseases, including cancer [43], genetic predisposition plays a crucial role, particularly in an individual’s ability to metabolize and synthesize specific components that mediate and contribute to inflammation, such as PUFAs [44]. Considering that our genetic potential is unchangeable, dietary interventions may influence the epigenome, thereby optimizing nutrigenetic outcomes. Bioactive compounds from food could affect gene expression through different pathways, including altering chromatin structure (by DNA methylation and histone modifications), modulating non-coding RNA, activating transcription factors through signaling cascades, and by direct interaction—binding to nuclear receptors. In addition, considering the established relationship between oxidative stress and abrasion of telomeres, it is likely that intake of antioxidant-rich foods, including omega-3 fatty acids, may offer significant health benefits [45]. A diet enriched with omega-3 fatty acids has been shown to modulate PUFA concentrations and their metabolites [46], contributing to a reduction in tumor growth and progression while promoting apoptotic pathways in certain experimental models [47,48]. Moreover, a lower omega-6/omega-3 ratio has been extensively linked to anti-inflammatory effects and reduced cancer risk, as it directly influences lipid-derived mediators, such as prostaglandins and leukotrienes, which are implicated in carcinogenesis [48]. Notably, SNPs in the *FADS* genes are associated with variations in circulating and tissue levels of EPA and DHA [49]. Consequently, specific genetic variants that result in an unfavorable omega-6/omega-3 ratio, may increase cancer susceptibility in individuals carrying such polymorphisms.

This review examines the existing literature on *FADS1/2* SNP variations in cancer patients, focusing on their impact on PUFA metabolism, concentrations in both blood and malignant tissues, and the potential implications for disease risk. Additionally, we explore how supplementation with anti-inflammatory PUFAs and their bioavailability in circulation and tissues may be influenced by *FADS* genetic variants.

### 3.1. Omega-3 FA Intervention and FADS1/2 in Patients with Cancer

It has been proven that flaxseed supplementation can reduce omega-6 PUFA levels in blood [50] and tissues [51,52], while data on its effect on increasing omega-3 LC-PUFAs are inconsistent. Table 1 highlights the sole interventional study included in this review—a randomized control trial which assessed the effect of flaxseed supplementation (30 g/day) on prostatic ALA levels, SNPs, and prostate cancer-specific biomarkers [32]. The study involved 134 men scheduled for prostatectomy, divided into two groups: one received 30 g of flaxseed daily and a control group maintained their usual diet for approximately 31 days prior to surgery. The researchers assessed dietary intake using the NCI Diet History Questionnaire alongside collecting blood, urine, anthropometric, and medical data from participants who were randomized based on race (Black vs. non-Black) and biopsy Gleason sums (<7 vs. ≥7). The researchers examined associations between selected SNPs (rs99780, rs174537, rs174545, rs174572, rs498793, rs3834458, and rs968567), prostatic ALA levels, and prostate cancer biomarkers. The findings indicated that, despite significantly higher dietary intake of ALA and total omega-3 fatty acids in the flaxseed group, there were no significant differences in prostatic ALA levels between the two groups. Additionally, no associations were observed between the targeted SNPs and prostatic ALA levels. Notably, prostatic ALA was positively associated with biomarkers of aggressive prostate cancer (prostate-specific antigen (PSA) and tumor proliferation rates), suggesting that prostatic ALA metabolism may be linked to disease aggressiveness. The study also noted that higher levels of EPA in prostatic tissue, resulting from ALA conversion, were not associated with reduced PSA levels or tumor proliferation rates.

In summary, this study [32] demonstrated that among prostate cancer patients, prostatic ALA levels were significantly and positively associated with biomarkers of aggressive disease, independent of dietary intake. This study also highlighted that genetic variation (rs498793) related to ALA metabolism may influence the association between ALA and prostate cancer.

This study is limited by its small sample size, short intervention period, lack of baseline tissue samples, and its secondary analysis design, which was not originally intended to evaluate gene–nutrient interactions. However, given that only one study met the inclusion criteria for this segment of the review, it is imperative to underscore the necessity for future research on omega-3 fatty acids and GLA supplementation in cancer patients, particularly in those with breast cancer who exhibit low levels of omega-3 fatty acids in the context of *FADS1/FADS2* gene polymorphisms [38]. This could explain the different response to omega-3 and GLA supplementation in breast cancer patients undergoing chemotherapy [46]. Therefore, investigating the interplay between omega-3 supplementation and *FADS1/FADS2* genotypes in breast cancer could provide valuable insights into personalized dietary interventions and therapeutic strategies.

### 3.2. PUFA Status and FADS1/2 in Patients with Cancer

Table 2 presents two cross-sectional and one case–control study investigating the interplay between fatty acid metabolism and *FADS1/FADS2* genes variants in patients with cancer. Desaturase enzymes regulate the synthesis of PUFAs and MUFAs, which are key regulators of membrane fluidity, inflammation, and cell signaling. Two studies [33,34] examined fatty acid metabolism in prostate cancer, while the third one focused on glioblastoma multiforme (GBM) [35]. One study investigated the impact of genetic variations in *FADS1/2* on circulating fatty acids [33], and another analyzed desaturase expression in both tumor tissue and peritumoral regions of GBM [35]. The third study examined desaturase expression only in tumor tissues [34].

A case–control study analyzed 24 circulating fatty acids in 2934 participants, including 1431 prostate cancer cases (585 Ghanaians, 407 African Americans, and 439 European Americans) and 1503 controls (658 Ghanaians, 381 African Americans, and 464 European Americans) [33]. The study revealed significant population differences in circulating levels of fatty acids, with Ghanaian men exhibiting the highest omega-3 and lowest omega-6 levels, leading to the lowest omega-6/omega-3 ratios, potentially contributing to delayed disease progression in this population. *FADS1/2* polymorphisms were found to significantly influence omega-3 and omega-6 fatty acid levels, with population-specific effects. In European American men, specific SNPs were associated with omega-6 (DGLA, DPA, and GLA), whereas no such associations were observed in African American and Ghanaian men. Notably, higher DHA, DPA, and EPA levels were inversely associated with prostate cancer risk in Ghanaian men [33], while omega-6 fatty acids were positively associated with prostate cancer in European Americans [33,34]. These findings are consistent with a previous study [53], which also reported that *FADS1/2* polymorphisms significantly influence omega-6 fatty acid levels, with population-specific differences and stronger genetic associations observed in European Americans compared to African Americans. Additionally, it was found that elevated levels of trans fatty acids (elaidic, palmitelaidic, and linoelaidic acids) were linked to an increased risk for prostate cancer across all groups, with Ghanaian men exhibiting the lowest average trans fatty acid levels [33].

Prostate cancer was also investigated, highlighting confirmed differences between African Americans and European Americans [34]. The study analyzed prostate tissue from 60 patients undergoing radical prostatectomy to assess PUFA status and genotype variations. The rs174537 genotype significantly influenced both omega-3 and omega-6 LC-PUFAs in tumor tissue, the G allele being associated with higher AA, ADA, DPA, and DHA levels and lower LA and DGLA levels—promoting cancer cell growth. Additionally, ADA and omega-3 fatty acids were positively associated with the G allele, while LA and DGLA showed a negative correlation. AA was the predominant LC PUFA in all samples, but its levels were strongly influenced by genotype. Additionally, ADA and omega-3 PUFA levels were positively associated with the G allele, while LA and DGLA showed a negative correlation [34]. A more recent Mendelian randomized study analyzed genetic instruments for PUFA metabolism in 46,155 cases and 270,342 controls. It found an association between AA levels and increased colorectal cancer risk but did not support the hypothesis that PUFAs lower cancer risk and mortality [54]. Methylation at cg27386326, within the *FADS* gene cluster, was closely linked to FADS1 activity and PUFA metabolism, showing an inverse correlation with AA levels and biosynthetic efficiency. Tumor-driven metabolic reprogramming affects PUFA desaturation pathways, influencing cancer progression and potential therapeutic targeting. These genetic and epigenetic factors contribute to population-specific differences in PUFA composition, particularly between African Americans and European Americans [34].

Another case–control study examined the expression of desaturases (FADS1, FADS2, and SCD) in glioblastoma tumors from 28 patients [35]. The authors observed that FADS2 expression was significantly lower in the tumor tissue compared to the peritumoral area, suggesting tumor-specific metabolic adaptation. Additionally, a coordinated regulation between FADS1 and FADS2 was noted in response to the tumor microenvironment [35]. These findings align with a more recent study that reported that elevated trans and omega-6 fatty acid levels promote prostate cancer, with *FADS1/2* genetic variations influencing susceptibility across different populations [33]. A previous study demonstrated that glioblastoma tumors downregulate FADS2 expression, potentially mitigating oxidative stress or reallocating metabolic resources [35]. Similarly, the study highlighted the role of SCD in tumor metabolism, noting its reduced expression in tumor regions compared to peritumoral areas. Palmitoleic acid (an MUFA produced by SCD) was identified as being positively associated with prostate cancer aggressiveness [33]. In contrast, reduced SCD expression was observed in glioblastoma tumors, which increased under nutrient-deficient conditions in glioblastoma cells [35]. Both studies underscore the role of desaturases in tumor metabolism, suggesting that SCD-mediated MUFA biosynthesis may have distinct functions in various cancers through genetic and epigenetic regulation, potentially influencing cancer progression [34,35].

Collectively, these studies provide valuable insights into the relationship between genetic factors and fatty acid metabolism in cancer development. Fatty acid levels, particularly omega-3 and omega-6, may influence the progression of cancer and risk across different populations. However, severe limitations, such as small sample sizes, which limit the applicability of findings and the statistical power needed to detect gene–nutrient interactions [33,34,35]; the lack of dietary PUFA intake data, which hinders the ability to model gene–diet interactions accurately [33,34]; and population heterogeneity, including demographic and lifestyle differences [33], highlight the need for further research. Such studies are essential to better understand mechanisms underlying fatty acid metabolism and tumor-specific adaptations, particularly at the epigenetic level.

### 3.3. PUFA Intake and Status, FADS1/2 Genotypes, and Potential Risk Factors for Cancer

A Mediterranean diet, rich in fruit, vegetables, and fiber, may reduce cancer risk, while a diet high in saturated fats and red meat may increase the risk [55]. Several studies have investigated the relationship between PUFA intake and cancer risk, highlighting the complex interplay between diet, genetics, and cancer development. In a case–control study, 305 oral cancer patients and 579 cancer-free individuals aged 20 to 80 years were categorized based on their fish consumption into three groups: 0-2 times/week, 3-6 times/week, and ≥7 times/week. The study found a significant interaction between the *FADS1* gene and fish intake in relation to oral cancer risk. Notably, individuals who consumed fish ≥7 times/week exhibited a 73% reduction in oral cancer risk compared to those with a fish intake of 0-2 times/week, suggesting a dose–response relationship [41]. The anti-inflammatory properties of LC-PUFAs, such as EPA and DHA, in fish, are believed to suppress inflammation and oxidative stress, thereby reducing cancer risk [41,55].

Similarly, a more recent case–control study with 1464 participants (402 gastric cancer cases and 1062 controls) investigated the relationship between dietary PUFA intake and gastric cancer risk. Using a semi-quantitative food frequency questionnaire, the researchers found that higher dietary intake of DHA was associated with a significant reduction in gastric cancer risk [42]. Conversely, participants with gastric cancer exhibited higher intake of omega-6 PUFAs, such as AA, as well as omega-3 ALA, compared to controls. Additionally, a combined higher intake of marine-derived fatty acids, EPA and DHA, showed a significant borderline trend toward reduced gastric cancer risk. Conversely, the highest tertile of AA intake showed a tendency to increase the risk of gastric cancer [42].

Two studies investigated the impact of fish oil supplementation on colorectal adenoma patients [36,40]. The first study, a randomized controlled trial, administered 2.5 g of fish oil daily to participants aged 40 to 80 years with a history of colorectal adenomas. Over six months, the results indicated no significant anti-proliferative effect or pro-apoptotic effect on rectal mucosal cells, suggesting that omega-3 supplementation at this dosage may not confer protective effects against colorectal cancer precursors. Notably, the 2.5 g/day dose was lower than those in some other trials, highlighting the complex interplay between genetics and dietary interventions [36,40]. No influence on RBC membrane omega-3 LC-PUFA percentages in response to supplementation was observed.

In one study, after six months, both healthy eating and Mediterranean diet groups significantly reduced SFA intake and increased omega-3 PUFA intake [37]. The healthy eating group adhered to the Healthy People 2010 diet guidelines, emphasizing balanced servings of fruits, vegetables, grains, and limited fats. In contrast, the Mediterranean group focused on high-omega-3 foods such as fish and flaxseed, increased MUFAs, and reduced omega-6 PUFAs. These dietary adjustment changes were reflected in serum fatty acids profiles; however, the only significant change was an increase in omega *n*-3 PUFA levels, suggesting that tissue-specific mechanisms may limit the impact of dietary modifications on colon fatty acid composition [37]. Other lifestyle factors also differed between cases and controls. Smoking prevalence was significantly higher among cases [41]. Additionally, cases had greater alcohol consumption, measured in grams of ethanol per day, and were less likely to engage in regular physical exercise [42].

Cyclooxygenase (COX 1 and COX-2) enzymes catalyze prostaglandin (PG) production, using EPA and AA as substrates. AA, released from membrane phospholipids, is converted by COX into the unstable PGH_2_ [56]. Synthases then produce PGE2, a bioactive lipid linked to inflammation and cancer progression [57,58]. PGE2 acts via prostaglandin E (EP) receptors, a subclass of G-protein-coupled receptors (GPCRs) [59]. Binding to EP2 and EP4 receptors—both of which are linked to Gαs proteins—activates adenylate cyclase, increasing cAMP and triggering protein kinase A (PKA)-mediated phosphorylation transcription of transcription factors like CREB, enhancing gene transcription. Both receptors also activate the GSK3β/β-catenin pathway, promoting cancer-related genes (such as c-myc, cyclin D1, and VEGF). EP4, unlike EP2, engages PI3K/Akt pathway signaling, aiding cancer cell proliferation and survival [60,61]. On the other hand, EPA and DHA have also been shown to influence COX activity and exhibit anti-cancer properties by downregulating COX expression, which leads to reduced synthesis of PGE2. Also, these fatty acids promote apoptosis by modulating Bcl-2 family proteins and suppressing VEGF signaling pathways [62]. It was found that urinary PGE metabolite (PGE-M) is associated with obesity, smoking, and lung metastases in breast cancer patients [63]. COX-2 is implicated in tumor growth, angiogenesis, and immune evasion [64]. Notably, individuals with the GG allele in *FADS1* variant rs174535 exhibited fewer COX-2-positve cells than GT/TT carriers, even after omega-3 PUFA or olive oil supplementation [36,40].

Fish oil (FO) supplementation has demonstrated effects on inflammatory markers in multiple studies [65,66,67]. Two studies included in this review [36,40] found that FO supplementation, rich in EPA and DHA, significantly reduced urinary PGE-M levels compared to olive oil, which primarily contains oleic and LA [68]. Increased urinary PGE-M correlated with higher rectal PGE2 levels. During the study, RBC membrane *n*-3 LC-PUFA levels rose by 76% in the FO group versus 6% in the olive oil group, while RBC membrane AA levels significantly declined in the FO group [36,40]. Despite its influence on AA levels, the *FADS1* polymorphism rs174537 did not modify FO effects [40]. A study using personalized omega-3 doses linked higher serum EPA:AA ratios to reduced colonic mucosal PGE2 levels [69]. These findings align with a previous study that reported that higher fish intake elevates LC-PUFAs, which could offer protection against inflammation and cancer [41]. While fish oil supplementation alters fatty acid composition, its impact on cancer-related inflammation is likely influenced by genetics and baseline inflammatory status [36].

These studies underscore the nuanced relationship between PUFA intake and cancer risk, influenced by factors such as genetic variations, specific cancer types, and dietary sources of PUFAs. While increased consumption of marine-derived omega-3 PUFAs appears to be beneficial in reducing the risk of certain cancers like oral and gastric cancer, supplementation alone may not yield similar protective effects against colorectal cancer precursors. This highlights the importance of considering dietary patterns, genetic predispositions, and cancer-specific mechanisms when evaluating the role of PUFAs in cancer prevention. In addition, these studies also have limitations which prevent drawing a firm conclusion. The generalizability of the findings was limited by small sample sizes and short intervention durations [36,37,40]. The reliance on self-reported dietary intake introduced recall bias [37,42], while PUFA levels were often assessed in serum or diet, such that more precise tissue-level measurements were lacking [36,42]. Focus on only a few SNPs, missing genetic variation [41,42], lack of adjustment for demographic differences like ethnicity and physical activity [36], and the absence of inflammatory biomarker data limited insight into mechanisms [36,42]. Addressing these limitations in future research would improve both the accuracy and interpretability of results.

### 3.4. Genetic Variation in the FADS1 Gene and Its Role in Cancer Risk

It was found that the *FADS1* rs174549 AA genotype significantly lowers risk for oral cancer, especially in male smokers and alcohol consumers aged ≤ 60 years, while this trend was not observed in GG carriers. Frequent fish consumption may further reduce risk in those with the mutant rs174549 genotype [41]. Similar results were reported for the *FADS1* rs174535 variant. In a fish oil supplementation study, this genetic variation did not affect RBC membrane omega-3 LC-PUFA levels but significantly influenced AA concentrations, highlighting the role of genetics in dietary response [36]. Another study confirmed that fish oil supplementation reduced RBC membrane AA contents [40]. Elevated AA and low omega-3 levels create a pro-inflammatory environment linked to breast cancer development [38]. It was found that the GG allele of rs174548 was associated with reduced FADS1 expression (but not FADS2) and that omega-6 PUFAs contribute more to lung cancer risk than omega-3, with a stronger protective effect observed in women. This identifies rs174548 as a potential novel genetic marker associated with lung cancer risk [39].

ALA impacts breast cancer by influencing proliferation pathways and regulating pro- and anti-apoptotic proteins. It inhibits ERBB1/EGFR, PTP1B, SHP2, and IGF-1R—proteins that suppress ERK signaling via Src kinase and limit ERK and Akt phosphorylation to restrain growth signaling [70]. In *FADS1* GT/TT carriers, ALA levels were linked to ER-positive breast cancer, while DPA was found in GG genotype with ER-negative cases. Across all genotypes, higher omega-3 levels in GG carriers correlated with lower breast cancer risk. Individuals carrying GT/TT with reduced fatty acid conversion may benefit more from increased omega-3 intake [38].

A study on gene–diet interaction analyzed AA concentrations in colon mucosae after six months on a Mediterranean or healthy diet (rich in fruits, vegetables, and whole grains and limiting saturated and total fat) [37]. Four SNPs (rs3834458, rs174556, rs174561, and rs174537) were genotyped [37]. Subjects on a Mediterranean diet with all major alleles for the *FADS1/2* genes had 16% lower AA concentrations in the colon mucosa, suggesting a reduced colon cancer risk due to lower PGE2 production [37]. Conversely, those without minor alleles had higher AA levels in serum. *FADS1/2* polymorphisms did not affect EPA concentrations in serum and colon tissue, likely due to dietary influence [37]. These findings highlight the role of diet-related PUFA intake in colon cancer prevention [39].

This review underscores the absence of definitive evidence regarding the impact of dietary patterns on desaturase activity and/or expression. While genetic variations in the *FADS1* and *FADS2* genes are linked to PUFA metabolism and cancer risk, the interplay between dietary PUFA intake and genetic factors remains insufficiently understood. Future research is needed to investigate the gene–nutrient interactions and their role in cancer development.

### 3.5. Strengths and Limitations

This study provides a systematic and up-to-date review of the relationship between PUFA metabolism, genetic variations in *FADS* genes, and cancer. It highlights the importance of gene–diet interactions in cancer research and identifies key areas for future investigation. Furthermore, the inclusion of multiple reputable databases ensures a thorough and comprehensive literature search.

This review is limited by the small number of included studies (*n* = 11), which may not offer a comprehensive overview of the topic. Additionally, the heterogeneity in study designs, sample sizes, and methodology makes it difficult to draw definitive conclusions. The lack of intervention studies further limits the ability to establish causal relationships between dietary PUFA intake, genetic variations, and cancer risk.

## 4. Materials and Methods

### 4.1. Scoping Review Approach

A scoping review approach was employed to explore a complex issue that has not been comprehensively reviewed to date and to highlight the existing knowledge gaps in the field.

### 4.2. Search Strategy

The PICOTSS framework—Population, Intervention (or Exposure), Comparator(s), Outcome(s), Timing, Setting(s), and Study Design—was used in this study.

Target population: Adults with different types of cancer.

Exposure: Omega-3 fatty acids, gamma-linolenic acid, fish oil, and foods rich in omega-3 fatty acids.

Comparators: Studies with comparable controls and polymorphism.

Outcome variables: *FADS1/FADS2* polymorphism in relation to fatty acid status or intake in cancer patients or in individuals with higher risk for cancer.

Timing: From inception to the end of 2024.

Settings: All settings included where cancer patients spent time (home, hospital, nursing home, etc.).

Study design: Observational, including cross-sectional and prospective studies and intervention studies

The search in the PubMed, Scopus, and Web of Science databases was conducted to examine relevant articles published from inception to the end of 2024. The first search examined articles published from inception to April 2024, and the updated search examined articles from May to December 2024. In the search strategies (strings adapted when necessary to fit the specific search requirements of each database), the following keywords and terms were used: (Nutrigenetic OR Nutrigenomic OR FADS1 OR FADS2 OR Fatty Acid Desaturase 1 OR Fatty Acid Desaturase 2 AND (omega-3 OR *n*-3 OR eicosapentaenoic acid OR EPA OR docosahexaenoic acid OR DHA OR fish oil OR fatty fish OR sea foods OR gamma-linolenic acid OR GLA OR evening primrose oil OR borage oil OR black currant oil OR spirulina OR purslane OR chia seeds OR flaxseed OR walnuts OR hemp seed oil) combined with cancer as the outcome term.

### 4.3. Eligibility Criteria

A comprehensive selection process was applied to identify relevant studies. Only research conducted on human participants over the age of 18 and published in English was considered, with no restrictions on publication date, concluding at the end of 2024. Two of the co-authors (V.Z., and M.P.) screened titles and abstracts according to these criteria, selecting eligible studies for further review. Full-text articles were reviewed by three co-authors (V.Z., M.P., and D.R.), and 20% were double-checked for accuracy. Reviews, book chapters, conference abstracts, letters, articles lacking usable data, and studies focused on children, adolescents, pregnant women, animal models, or cell cultures were excluded. Studies involving participants with chronic conditions (e.g., kidney or liver disease, HIV, thyroid disorders, or glucocorticoid use) were also excluded. Additionally, in order not to miss relevant articles, the reference lists of the related articles and reviews were examined.

The final selection of articles was based on the following criteria: (1) observational or epidemiological studies, (2) clinical trials focused on interventions related to omega-3 fatty acid intake and/or gamma linolenic acid intake, and (3) studies on genetic and epigenetic variations in the *FADS* gene cluster revealing complex gene–diet supplementation interactions. Any disagreements during the selection process were resolved through consensus among all the authors.

### 4.4. Data Extraction

The findings from the full texts of eligible papers were summarized in tables based on the main categories of the outcome measures. The following information was extracted from the included articles and presented in the tables: name of the first author; publication year; country; cancer area; study design, sample size and sex of participants; age range or mean age; genetic variation in the *FADS1/FADS2* gene cluster; dietary assessment; PUFA status (blood and tissue); intervention/supplementation and duration (Table 1); associations between *FADS1/FADS2* and PUFA status (Table 2); gene–PUFA interactions; and cancer risk (Table 3).

## 5. Conclusions

Current evidence underscores the complex relationship between genetic polymorphisms, fatty acid metabolism, and cancer development. Variants in the *FADS1* and *FADS2* genes may influence blood and tissue levels of LC-PUFAs, potentially shifting the balance toward more favorable anti-inflammatory omega-3 pathways. Notably, lower levels of omega-3 LC-PUFAs and elevated levels of omega-6 LC-PUFAs have been observed in cancer patients and malignant tissue, suggesting a potential link to disease progression. To date, the most consistent association identified is between the rs174537 variant and altered PUFA metabolism in prostate and breast cancer. While initial studies suggest that omega-3 supplementation may improve LC-PUFA profiles, data on the interaction between *FADS1*/*FADS2* polymorphisms and therapeutic response remain limited. Only one study has examined omega-3 supplementation in relation to *FADS* gene variants in prostate cancer, and, to date, no studies have evaluated the role of GLA supplementation. These gaps underscore the need for further research into the molecular mechanisms—especially epigenetic modifications—linking genetic variation to cancer progression. Genome-tailored nutritional interventions hold promise as a novel strategy to enhance anti-inflammatory responses and improve outcomes in genetically defined cancer subgroups.

## 6. Current Problems and Future Directions

Key challenges include the absence of large-scale, well-designed clinical trials and limited understanding of how genetic variability affects fatty acid metabolism and treatment outcomes in cancer. The lack of translational research bridging laboratory findings and clinical applications also hinders progress. Additionally, the influence of environmental and dietary factors on gene–nutrient interactions remains poorly understood. Future research should prioritize mechanistic studies—particularly at the epigenetic level—and conduct genome-informed nutritional intervention trials assessing the therapeutic potential of omega-3 and GLA supplementation in stratified patient populations. Advancing this field will require the integration of nutrigenomics into clinical oncology, implementation of precision nutrition strategies based on individual genetic profiles, and interdisciplinary collaboration among geneticists, nutritionists, oncologists, and bioinformaticians to develop personalized dietary approaches that optimize cancer treatment outcomes.
